# Single-Cell *in Situ* RNA Analysis With Switchable Fluorescent Oligonucleotides

**DOI:** 10.3389/fcell.2018.00042

**Published:** 2018-04-11

**Authors:** Lu Xiao, Jia Guo

**Affiliations:** Biodesign Institute and School of Molecular Sciences, Arizona State University, Tempe, AZ, United States

**Keywords:** transcriptomics, genomics, fluorescence *in situ* hybridization, strand displacement reactions, RNA expression, oligonucleotides, fluorescent probes, single-cell

## Abstract

Comprehensive RNA analyses in individual cells in their native spatial contexts promise to transform our understanding of normal physiology and disease pathogenesis. Here we report a single-cell *in situ* RNA analysis approach using switchable fluorescent oligonucleotides (SFO). In this method, transcripts are first hybridized by pre-decoding oligonucleotides. These oligonucleotides subsequently recruit SFO to stain their corresponding RNA targets. After fluorescence imaging, all the SFO in the whole specimen are simultaneously removed by DNA strand displacement reactions. Through continuous cycles of target staining, fluorescence imaging, and SFO removal, a large number of different transcripts can be identified by unique fluorophore sequences and visualized at the optical resolution. To demonstrate the feasibility of this approach, we show that the hybridized SFO can be efficiently stripped by strand displacement reactions within 30 min. We also demonstrate that this SFO removal process maintains the integrity of the RNA targets and the pre-decoding oligonucleotides, and keeps them hybridized. Applying this approach, we show that transcripts can be restained in at least eight hybridization cycles with high analysis accuracy, which theoretically would enable the whole transcriptome to be quantified at the single molecule sensitivity in individual cells. This *in situ* RNA analysis technology will have wide applications in systems biology, molecular diagnosis, and targeted therapies.

## Introduction

The ability to profile a large number of distinct transcripts in single cells *in situ* is crucial for our understanding of cancer, neurobiology, and stem cell biology (Crosetto et al., 2014). The differences between individual cells in complex biological systems may have significant consequences in the function and health of the entire systems. Thus, single cell analysis is required to explore such cell heterogeneity. Due to the inherent complexity of gene expression regulatory networks, comprehensive molecular profiling is required to systematically infer the functions and interactions of different RNA species. The precise location of cells in a tissue and transcripts in a cell is critical for effective cell-cell interactions and gene expression regulation, which can determine cell fates and functions. Therefore, to fully understand the organization, regulation, and function of a heterogeneous biological system, highly multiplexed single-cell *in situ* RNA analysis is critically needed.

Next-generation sequencing (Guo et al., 2010; Metzker, 2010) and microarray technologies (Hoheisel, 2006) have been widely used to study gene expression regulation in health and disease by profiling RNA on a genome-wide scale. However, as transcripts are extracted, purified and then analyzed in these approaches, the RNA location information is lost. Imaging-based methods, such as molecular beacons (Guo et al., 2012; Huang and Martí, 2012), templated fluorescence activation probes (Franzini and Kool, 2009), and fluorescence *in situ* hybridization (FISH) (Raj et al., 2008), allow transcripts to be quantified in their native spatial contexts in single cells. Nonetheless, due to the spectral overlap of commonly available fluorophores, these methods can only detect a handful of different RNA species in one sample.

To enable comprehensive single-cell *in situ* RNA analysis, several approaches have been investigated. For instance, *in situ* sequencing (Ke et al., 2013; Lee et al., 2014) has been explored to enable transcriptome profiling in individual cells. However, this method has limited detection efficiency and may miss low-expression transcripts. Combinatorial labeling (Levsky et al., 2002; Lubeck and Cai, 2012; Levesque and Raj, 2013) and reiterative hybridization (Xiao and Guo, 2015; Guo, 2016; Shaffer et al., 2017; Mondal et al., 2018) offer single-molecule detection sensitivity, but these approaches suffer from limited multiplexing capacities. Recently, sequential hybridization (Lubeck et al., 2014; Shah et al., 2016) and multiplexed error-robust fluorescence *in situ* hybridization (MER-FISH) (Chen et al., 2015; Moffitt et al., 2016a,b) have been developed for highly multiplexed single-molecule RNA detection. In these methods, to stain the same RNA molecules in different analysis cycles, several approaches have been explored to remove the fluorescence signals at the end of each cycle. Such approaches include probe degradation by DNase, photobleaching, and disulfide based chemical cleavage. Nevertheless, probe degradation by DNase is limited by its low signal removal efficiency. In addition, DNase removes all the probes, including the large oligonucleotides library hybridized to their RNA targets. Consequently, this expensive oligonucleotides library has to be re-hybridized in every analysis cycle, which will increase the assay time and cost. Photobleaching erases fluorescence signals in different imaging areas sequentially. As a result, it is less time-effective and has low sample throughput. The disulfide based probes can cross-react with the endogenous thiol groups and the thiol groups generated by fluorophore cleavage in previous cycles, which will lead to high background and false positive signals.

Here, we report a single-cell *in situ* RNA analysis approach using switchable fluorescent oligonucleotides (SFO). In this method, RNA molecules are first hybridized by pre-decoding oligonucleotides, which subsequently recruit SFO to stain their RNA targets. After imaging, SFO are removed by strand displacement reactions. Upon continuous cycles of target staining, fluorescence imaging, and SFO removal, varied RNA species are identified by unique fluorophore sequences at the optical resolution. To demonstrate the feasibility of this approach, we show that the hybridized SFO can be efficiently removed by strand displacement reactions within the cellular environment in 30 min. We also demonstrate that this probe removal process maintains the RNA integrity and keeps the pre-decoding oligonucleotides hybridized to their RNA targets. Additionally, we show that RNA can be quantified with high accuracy in at least eight continuous hybridization cycles, which theoretically would allow the whole transcriptome to be profiled in individual cells *in situ*.

## Materials and methods

### General information

Chemicals and solvents were purchased from Sigma-Aldrich or Ambion and were used without further purification, unless otherwise noted. Biogreagents were purchased from Invitrogen, unless otherwise indicated.

### Cell culture

HeLa CCL-2 cells (ATCC) were maintained in Dulbecco's modified Eagle's Medium supplemented with 10% fetal bovine serum, 10 U mL^−1^ penicillin and 100 g mL^−1^ streptomycin in a humidified atmosphere at 37°C with 5% CO_2_. Cells were plated on chambered coverglass (Thermo Scientific) and allowed to reach 60% confluency in 1–2 days.

### Cell fixation

Cultured HeLa CCL-2 cells were first washed with 1 X PBS at room temperature for 5 min, fixed with fixation solution [4% formaldehyde (Polusciences) in 1 X PBS] at room temperature for 10 min, and subsequently washed another 2 times with 1 X PBS at room temperature, each for 5 min. The fixed cells were then permeabilized with 70% (v/v) EtOH at 4°C at least overnight.

### Probe design

The pre-decoding probes with a length of 70 nt contain three 20 nt sequences: (i) a target-binding sequence for *in situ* hybridization to the target RNA, and (ii) two repeated readout sequences for decoding hybridization. The three sequences are separated from each other by a flanking 5T spacer. The target-binding sequence was designed by the Stellaris Probe Designer provided by Biosearch Technology. The sequences of pre-decoding probes are provided in Table S1.

The decoding probe (SFO) with a length of 40 nt contains two 20 nt sequences: (i) a binding sequence complimentary to the readout sequence of the pre-decoding probes, and (ii) a toehold sequence for strand displacement reactions. The decoding probe is conjugated to fluorophores with the 5′-amino modification. The sequence of the decoding probe is provided in Table S1.

The eraser oligonucleotide with a length of 40 nt is complimentary to the decoding probe. The sequence of the eraser oligonucleotide is provided in Table S1.

The SFO-orthogonal oligonucleotide with a length of 40 nt is conjugated to fluorophores with the 5′-amino modification. The sequence of the SFO-orthogonal oligonucleotide is provided in Tabl S1.

To further ensure the specificity, all the sequences above were screened against the human transcriptome by using Basic Local Alignment Search Tool (BLAST) (Camacho et al., 2009) to ensure there were no more than 10 nt of homology. Sequence alignment test were also performed by BLAST within these sequences to ensure there were no more than 8 nt of homology.

### Probe preparation

Pre-decoding oligonucleotides belonging to one library (IDT) were mixed and then stored as pre-decoding probe stock solution (10 mM in 0.01X Tris EDTA, pH 8.0) at 4°C.

The 5′-amino modified decoding probe or the SFO-orthogonal oligonucleotide (IDT), at a scale of 1 nmol, was dissolved in 3 μL of nuclease-free water. To this solution was added sodium bicarbonate aqueous solution (1M, 3 μL) and Cy3 (AAT Bioquest) or Cy5 (AAT Bioquest) in DMF (20 mM, 5 μL). The mixture was incubated at room temperature for 2 h and then purified using a nucleotide removal kit (Qiagen). The fluorophore conjugated oligonucleotides were subsequently purified via an HPLC (Agilent) equipped with a C18 column (Aligent) and a dual wavelength detector set to detect DNA absorption (260 nm) and the fluorophore absorbtion (555 nm for Cy3, 650 nm for Cy5). For the gradient, triethyl ammonium acetate (Buffer A) (0.1 M, pH 6.5) and acetonitrile (Buffer B) (pH 6.5) were used, ranging from 7 to 30% Buffer B over the course of 30 min, then at 70% Buffer B for 10 min followed by 7% Buffer B for another 10 min, all at a flow rate of 1 mL min^−1^. The collected fraction was then dried in a Savant SpeedVac Concentrator and stored as decoding probe stock solution or SFO-orthogonal oligonucleotide stock solution at 4°C in 100 μL 0.01X Tris EDTA (pH 8.0).

The eraser oligonucleotide was dissolved and stored as displacement stock solution (10 mM in 0.01X Tris EDTA, pH 8.0) at 4°C.

### Pre-decoding hybridization

To 100 μL of pre-decoding hybridization buffer (100 mg mL^−1^ dextran sulfate, 1 mg mL^−1^ Escherichia coli tRNA, 2 mM vanadyl ribonucleoside complex, 20 μg mL^−1^ bovine serum albumin, and 10% formamide in 2 X SSC) was added 1 μL of pre-decoding probe stock solution. Then the mixture was vortexed and centrifuged to obtain pre-decoding hybridization solution.

HeLa CCL-2 cells after fixation and permeabilization were first incubated with wash buffer (2 mM vanadyl ribonucleoside complex and 10% formamide in 2 X SSC) for 5 min at room temperature, then incubated with 100 μL of pre-decoding hybridization solution at 37°C overnight. Cells were then washed three times with wash buffer, each for 30 min, at 37°C.

Cells were then post-fixed with post-fixation solution [4% formaldehyde (Polusciences) in 2X SSC] at room temperature for 10 min, and subsequently washed another three times with 2X SSC at room temperature, each for 5 min.

### Decoding hybridization

To 100 μL of decoding hybridization buffer (100 mg mL^−1^ dextran sulfate, 2 mM vanadyl ribonucleoside complex, and 10% formamide in 2 X SSC) was added 5 μL of decoding probe stock solution with or without 5 μL of SFO-orthogonal oligonucleotide stock solution. Then the mixture was vortexed and centrifuged to obtain decoding hybridization solution.

Cells labeled with pre-decoding probes were directly incubated with 100 μL of decoding hybridization solution at 37°C for 30 min, and washed once with wash buffer at 37°C for 30 min. After incubation with GLOX buffer (0.4% glucose and 10 mM Tris HCl in 2 X SSC) for 1–2 min at room temperature, the stained cells were imaged in GLOX solution (0.37 mg mL^−1^ glucose oxidase and 1% catalase in GLOX buffer).

### Displacement of decoding probes

To 100 μL of displacement buffer (100 mg mL^−1^ dextran sulfate, 2 mM vanadyl ribonucleoside complex, and 10% formamide in 2 X SSC) was added 5 μL of displacement stock solution. Then the mixture was vortexed and centrifuged to obtain displacement solution.

Cells after imaging were incubated with 100 μL of displacement solution at 37°C for 30 min, and washed 3 times with 1X PBS at 37°C, each for 15 min, then followed by the next cycle of decoding hybridization.

### Imaging and data analysis

Cells were imaged under a Nikon Ti-E epofluorescence microscope equipped with a 100X objective, using a 5 μm range and 0.3 μm z spacing. Images were captured using a CoolSNAP HQ2 camera and NIS-Elements Imaging software. Chroma filters 49004 and 49009 were used for Quasar 579 and Cy5, respectively.

Fluorescent spots in each hybridization cycle were identified and localized by SpotDetector (Olivo-Marin, 2002). For the detected FISH spots, their intensities in the Cy3 and Cy5 channels were compared to determine the color of the spots. Raw images of the same cells in different cycles of hybridization were aligned to the same coordination system established by the images collected in the first cycle of hybridization based on one specific spot reappearing in each cycle. Spots in the first hybridization cycle with the distance less than 2 pixels (320 nm) to those in the second hybridization cycle were extracted as the barcodes, which corresponded to a potential mRNA molecule. Spots in the following hybridization cycles that shared the distance less than 2 pixels (320 nm) with the barcodes were identified as the reappearance of the barcodes. And the barcode reappearance percentage in each hybridization cycle was then calculated.

## Results

### Platform design

**Figure 1:**
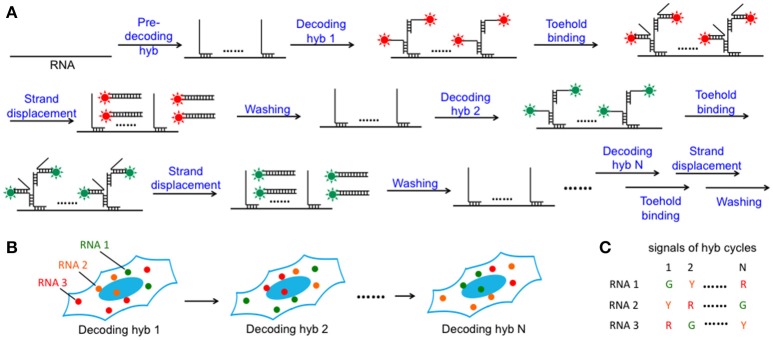
Highly multiplexed single-cell *in situ* RNA analysis with SFO. **(A)** Each transcript is first hybridized with a set of pre-decoding probes, which have varied target-binding sequences to hybridize to the different regions on the target RNA and the shared decoding sequence to recruit SFO as decoding probes. After imaging, the hybridized SFO is removed by strand displacement reactions. Through reiterative cycles of SFO hybridization, fluorescence imaging and strand displacement, the target RNA is sequentially stained by a set of SFO labeled with varied fluorophores. **(B)** Schematic diagram of the N cycles of hybridization images. In each cycle, individual transcript is visualized as a single spot with a specific color. **(C)** As RNA molecules remain in place during different hybridization cycles, different RNA species can be identified by the unique color sequences.

In this SFO-based RNA profiling approach (Figure 1), individual RNA target is first hybridized by a set of non-fluorescent pre-decoding oligonucleotides with varied target binding sequences. These oligonucleotides also have one or multiple decoding oligonucleotides binding sequences, which can recruit SFO as decoding probes. Each of the subsequent analysis cycles consists of three steps. First, SFO are hybridized to pre-decoding probes to stain the RNA targets. In the second step, fluorescence images are acquired with each RNA molecule visualized as a single spot. Finally, oligonucleotide erasers, which are perfectly complementary to SFO, are applied to remove SFO by strand displacement reactions (Zhang and Seelig, 2011). These oligonucleotide erasers hybridize to the toehold on SFO, branch migrate and release SFO from the pre-decoding probes. Through reiterative cycles of target staining, fluorescence imaging and SFO release, each transcript is identified by a fluorescence sequence barcode. With M fluorophores applied in each cycle and N sequential cycles, a total of M^N^ RNA species can be quantified in single cells *in situ*.

### SFO removal efficiency

**Figure 2:**
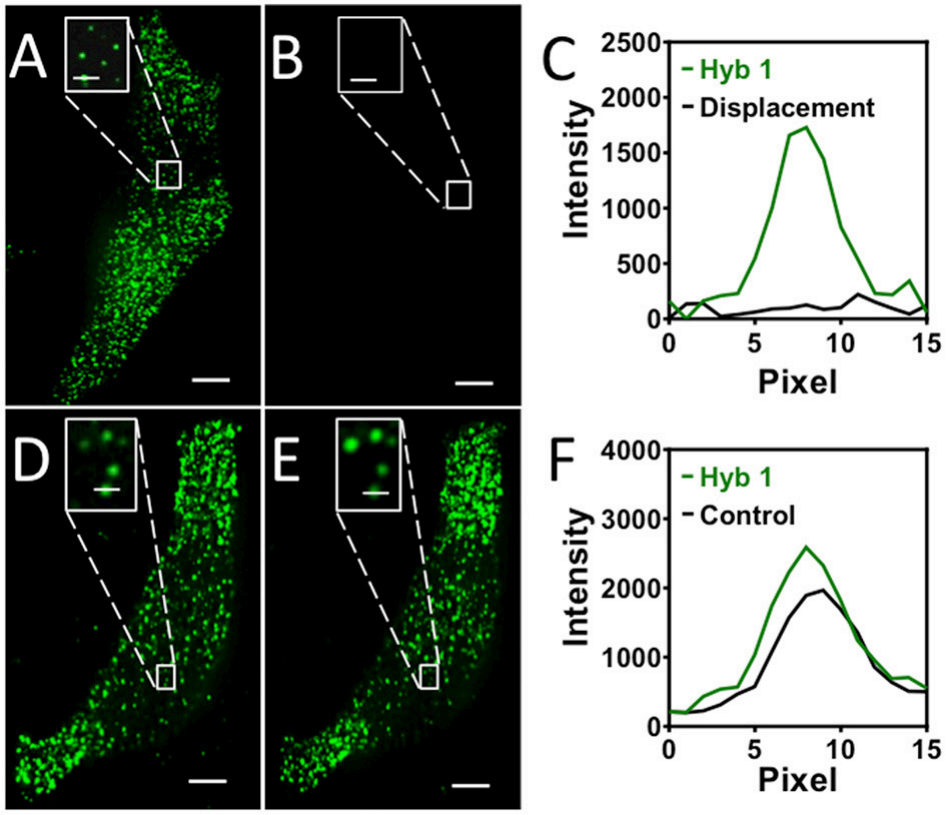
**(A)** GAPDH transcripts are stained by Cy3 labeled SFO. **(B)** SFO is removed by the eraser oligonucleotide. **(C)** Signal intensity profiles corresponding to the marked FISH spot in **(A,B)**. **(D)** GAPDH transcripts are stained by Cy3 labeled SFO. **(E)** The stained cells are incubated with an orthogonal oligonucleotide. **(F)** Signal intensity profiles corresponding to the marked FISH spot in **(D,E)**. Scale bars, 5 μm.

One requirement for the success of this SFO-based RNA profiling technology is that fluorescent decoding probes need to be removed very efficiently at the end of each analysis cycle. In this way, the minimized fluorescence signal leftover will not lead to false positive signals in the subsequent cycles. Additionally, the efficient removal of SFO will regenerate the single-stranded SFO-binding sequences on pre-decoding probes, so that SFO can be recruited in the following cycle to stain the target RNA again. To assess the SFO stripping efficiency, we stained mRNA GAPDH with Cy3 labeled decoding probes (Figure 2A). After incubating the stained cells with the oligonucleotide eraser for 30 min at 37°C, almost all the original FISH spots become undetectable (Figures 2B,C). We also performed control experiments by incubating the stained cells with an SFO-orthogonal oligonucleotide (Figure 2D). The fluorescence intensities of the Cy3 stained GAPDH remained largely the same before and after the oligonucleotide incubation (Figures 2E,F). These results indicate that SFO can be efficiently removed by strand displacement reactions.

### Effects of the strand displacement reactions

Another requirement for the success of this SFO-based approach is that the strand displacement reactions should maintain the RNA integrity, so that the same transcripts can be restained in the subsequent cycles. Additionally, it is preferred to keep the pre-decoding probes hybridized to their RNA targets throughout the assay, rather than to apply them in every analysis cycle. This is essential for the following reasons. First, due to the theoretical hybridization efficiency of ~75% (Lubeck and Cai, 2012), a small percentage of transcripts are not hybridized with enough pre-decoding probes to make them detectable. And these undetectable RNA can be different transcripts in different analysis cycles, if the pre-decoding probes are removed and rehybridized in each cycle. Consequently, many missing spots in the aligned fluorophore sequences will be generated, leading to the increased error rate. Furthermore, as the hybridization of the pre-decoding probes takes overnight to 36 h, it is time-consuming to apply this step in each cycle. Finally, for highly multiplexed RNA profiling, the pre-decoding probes library is usually composed of thousands of oligonucleotides. Thus, it will make the assay less cost-effective if the expensive pre-decoding library is removed and re-hybridized in every cycle.

**Figure 3:**
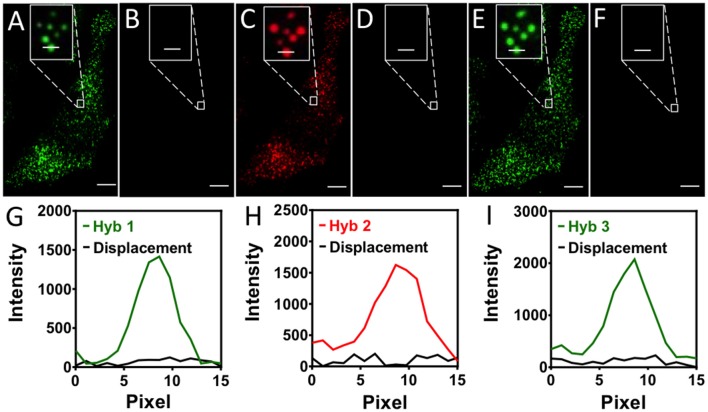
**(A)** In the first hybridization cycle, GAPDH transcripts are stained by Cy3 labeled SFO. **(B)** SFO is removed by the eraser oligonucleotide. **(C)** In the second hybridization cycle, GAPDH transcripts are stained by Cy5 labeled SFO. **(D)** SFO is removed by the eraser oligonucleotide. **(E)** In the third hybridization cycle, GAPDH transcripts are stained by Cy3 labeled SFO. **(F)** SFO is removed by the eraser oligonucleotide. **(G)** Signal intensity profiles corresponding to the marked FISH spot in **(A,B)**. **(H)** Signal intensity profiles corresponding to the marked FISH spot in **(C,D)**. **(I)** Signal intensity profiles corresponding to the marked FISH spot in **(E,F)**. Scale bars, 5 μm.

To assess the effects of the strand displacement reactions on the RNA targets and the hybridized pre-decoding probes, we stained mRNA GAPDH in three continuous hybridization cycles (Figure 3). In each cycle, Cy3 or Cy5 labeled SFO were applied to stain the transcripts, and were subsequently removed very efficiently using the same oligonucleotide eraser. We counted 1032 and 1045 spots in the first and second cycle, respectively. Among these spots, 803 spots were colocalized. These results are consistent with the ones obtained by using two sets of different colored FISH probes to stain the same transcripts (Raj et al., 2008). The small fraction of spots that did not colocalize may correspond to the non-specifically bound probes. To exclude these off-target signals, we define only the spots colocalized in the first two cycles as true mRNA signals. With our approach, 99% of the true signals reappeared in the third cycle. In comparison, when both pre-decoding and decoding probes are degraded using DNase, only 78% of spots reoccur in the third cycle (Lubeck et al., 2014). These results suggest that the DNA displacement reactions do not damage the RNA integrity, and the pre-decoding probes remain hybridized to their RNA targets throughout the assay. In this way, the analysis accuracy is improved and the assay time and cost are reduced.

### Eight-cycle RNA restaining

**Figure 4:**
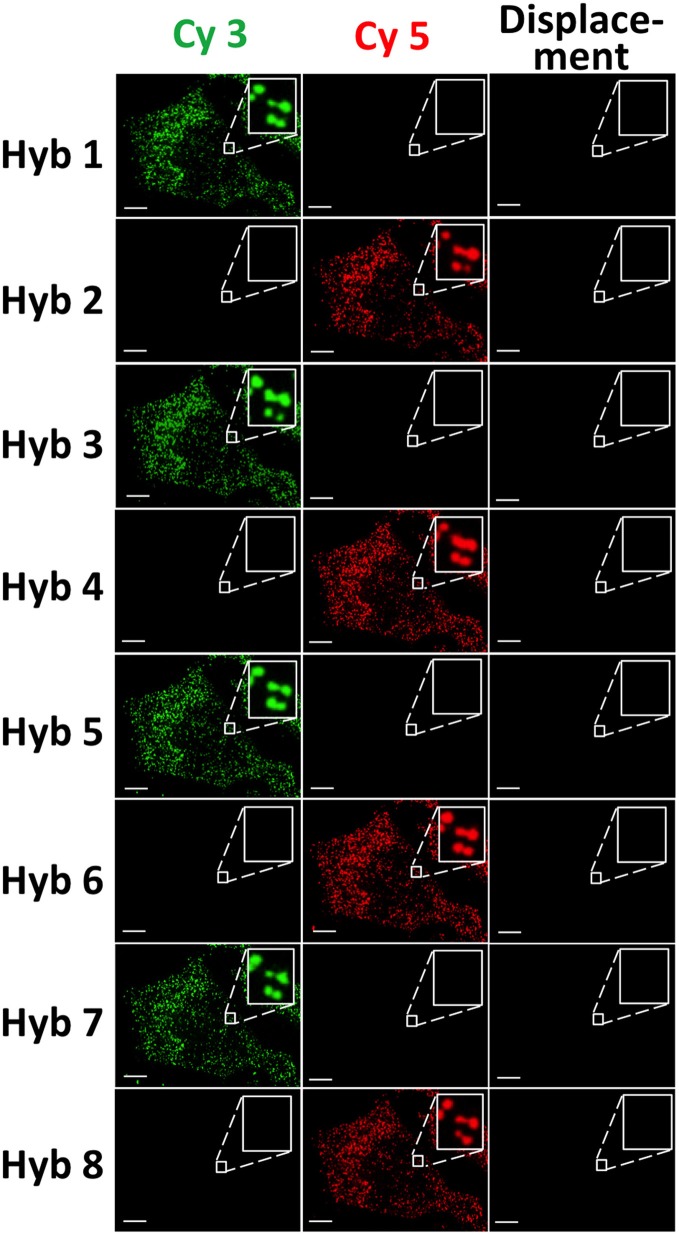
GAPDH transcripts are stained by SFO in eight consecutive hybridization cycles. In the odd cycles, cells are incubated with Cy3 conjugated SFO and a Cy5 labeled orthogonal oligonucleotide. In the even cycles, cells are incubated with Cy5 conjugated SFO and a Cy3 labeled orthogonal oligonucleotide. After target staining, images are captured in the Cy3 and Cy5 fluorescence channels. Following strand displacement reactions, images are captured in the Cy3 channel in the odd cycles and in the Cy5 channel in the even cycles. Scale bars, 5 μm.

**Figure 5:**
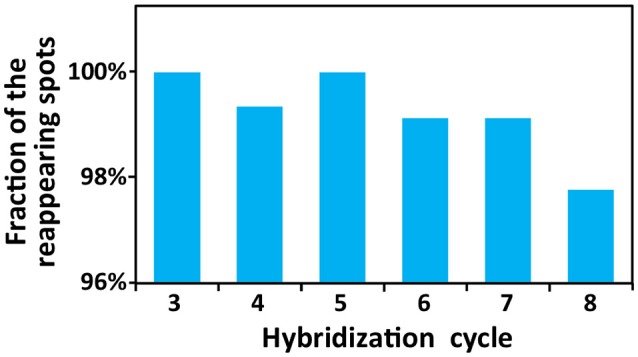
Fractions of the spots colocalized in the first two hybridization cycles that reappear in the following analysis cycles.

**Figure 6:**
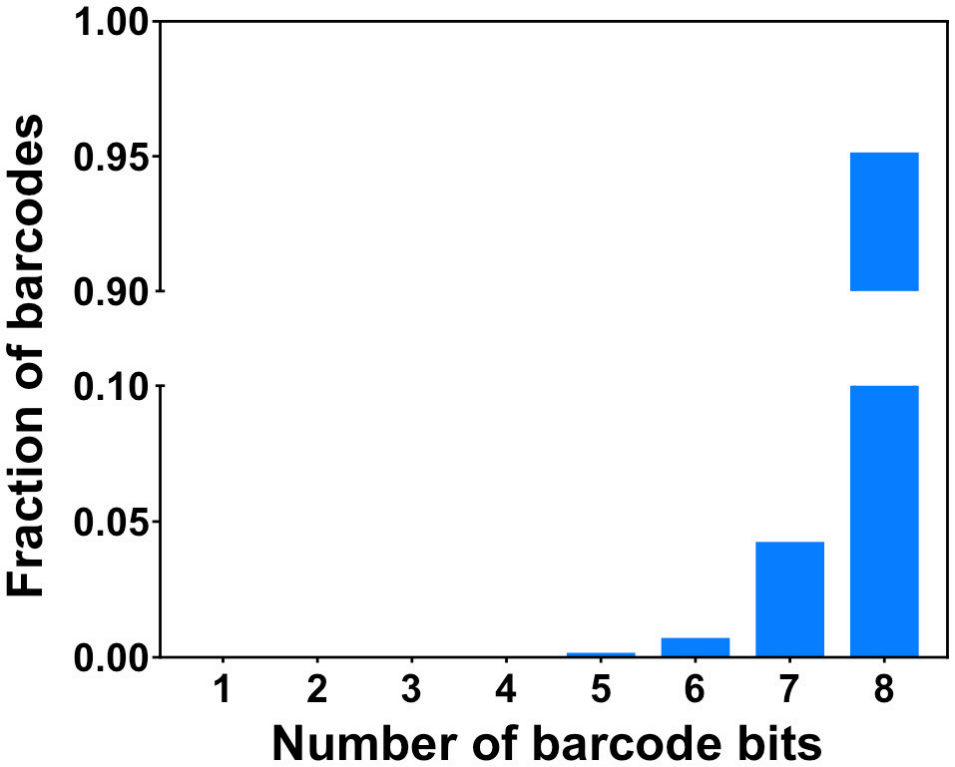
Fractions of the identified barcodes with different numbers of bits.

**Figure 7:**
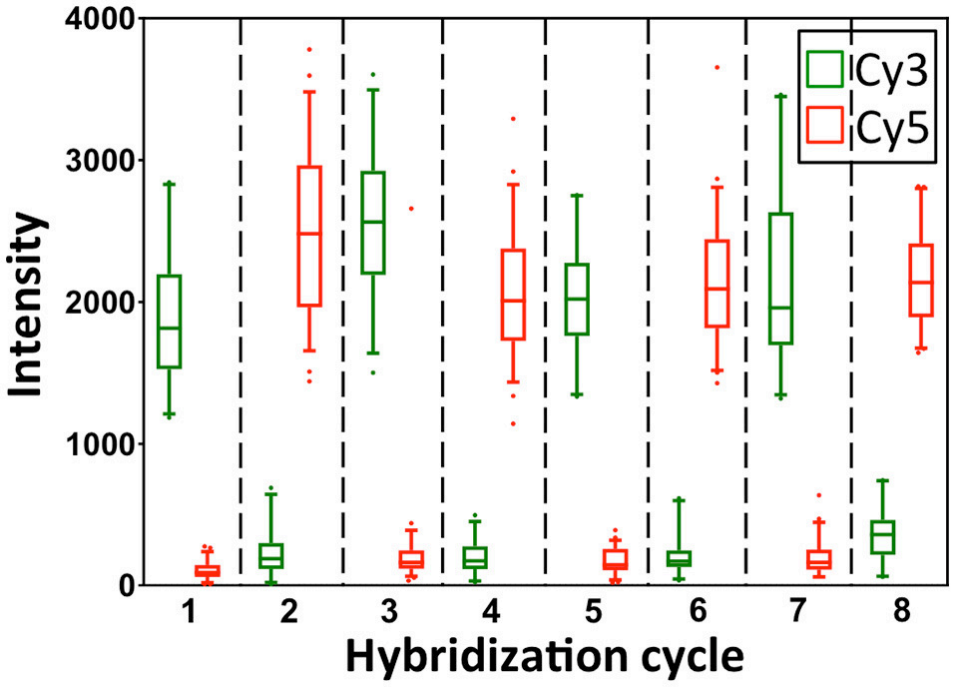
Intensity distributions of GAPDH FISH spots (*n* = 60 spots) in Cy3 and Cy5 channels over the eight hybridization cycles.

**Figure 8:**
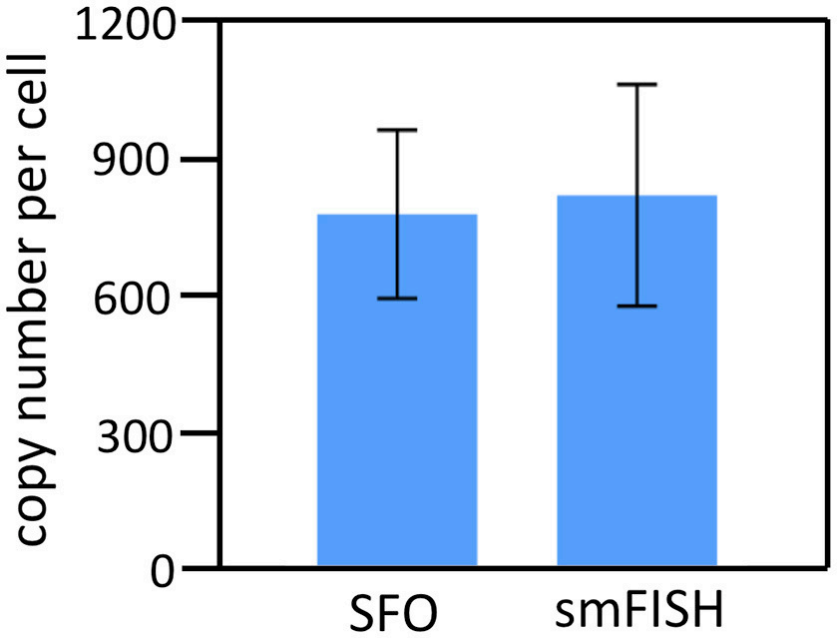
The GAPDH mean copy numbers per cell (*n* = 45 cells) obtained using the SFO-based approach and the conventional smFISH (*p* > 0.65).

To demonstrate the multi-cycle potential of our approach, we stained mRNA GAPDH in eight consecutive hybridization cycles using SFO (Figure 4). To evaluate the target staining specificity, we incubated the cells with Cy3 conjugated SFO together with a Cy5 labeled orthogonal oligonucleotide in the odd hybridization cycles, and with Cy5 conjugated SFO and a Cy3 labeled orthogonal oligonucleotide in the even cycles. In the first cycle, the FISH spots were only observed in the Cy3 channel, suggesting that mRNA GAPDH is specifically stained by the corresponding SFO. After signal detection and strand displacement reactions, we imaged the cells again to confirm the efficient stripping of SFO. This process of staining, imaging and stripping was repeated eight times to obtain the 8-bit fluorophore sequence barcode for the target mRNA. For the spots co-localized in the first two cycles (*n* = 1470), more than 97% of these spots reappeared in each of the following cycles (Figure 5). And over 95% of the spots were successfully identified in all the hybridization cycles (Figure 6). A plot of the signal intensities of the FISH spots in both the Cy3 and Cy5 channels vs. the hybridization cycles is shown in Figure 7. Due to the high staining specificity, all the FISH spots were unambiguously detected in the correct fluorescence channels. We also performed control experiments to stain mRNA GAPDH using the conventional smFISH method. The copy numbers per cell obtained by the two methods (Figure 8), together with those reported previously using RNA-Seq (Uhlén et al., 2015), are consistent with each other. These results suggest that transcripts can be quantitatively profiled in single cells *in situ* by multi-cycle staining using the SFO-based approach.

**Figure 9:**
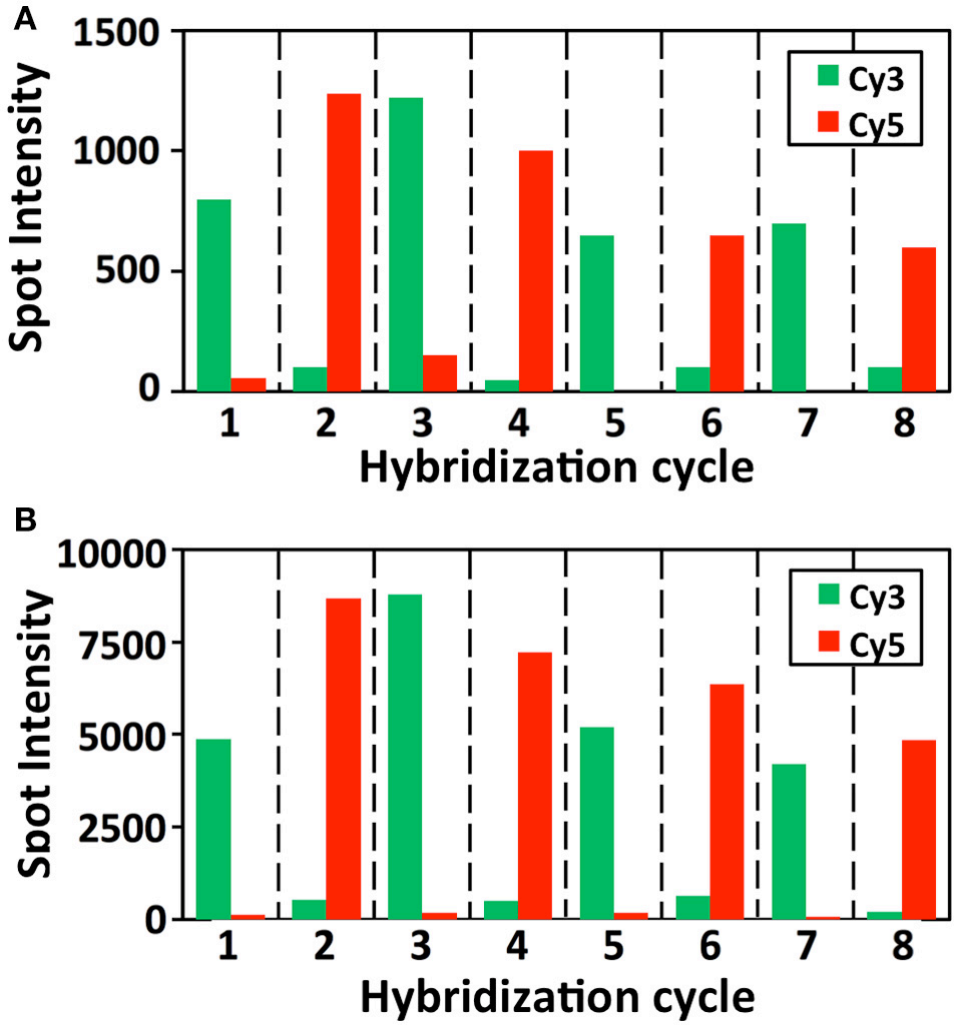
Signal intensities of **(A)** a weak and **(B)** a strong GAPDH FISH spot in Cy3 and Cy5 channels over the eight hybridization cycles.

In each cycle of MER-FISH, only certain transcripts are stained and other RNA targets remain unlabeled. Thus, to determine which transcripts are stained in a specific cycle, a detection threshold has to be manually selected by comparing the signal intensities of different FISH spots. However, due to the imperfect probe hybridization efficiency, RNA secondary structures, proteins bound to transcripts and other factors, even individual transcripts from the same RNA species can have significantly different staining intensities (Figure 7). As a result, the artificial detection threshold can lead to false negative signals, if the stained transcripts have low signal intensities. This threshold will also result in false positive signals, if the un-stained transcripts have high fluorescence intensities, which are generated as the signal leftovers from the previous cycles. In contrast, all the RNA targets are stained simultaneously in every cycle in the SFO-based approach. Rather than using a threshold to identify the stained transcripts, we compare the signal intensities of the same spot in different fluorescence channels to determine which SFO is hybridized to the specific RNA target. In this way, the correct fluorescence sequence can be unambiguously identified for both the weak spots (Figure 9A) and the strong spots (Figure 9B) in each analysis cycle. These results suggest that the SFO-based approach avoids the false positive and negative signals generated by the artificial threshold, and have enhanced detection sensitivity and analysis accuracy.

## Discusssion

We have developed an SFO-based technology for *in situ* RNA profiling. Compared with the existing methods, our approach has the following advantages. (i) By detecting transcripts directly without target sequence amplification, our technology enables RNA analysis at the single-molecule sensitivity. (ii) In this method, different RNA species can be distinguished by the varied color sequences, whose number increases exponentially with the number of hybridization cycles. Thus, our approach has the potential to enable highly multiplexed RNA analysis. (iii) All the distinct SFO in the whole specimen can be simultaneously removed by their corresponding eraser oligonucleotides. Therefore, our approach has high sample throughput, and allows a large number of cells to be quantified in a short time. (iv) As SFO can be very efficiently removed and have minimized cross-reactions with endogenous biomolecules and other probes, our approach has enhanced signal to noise ratio. (v) By keeping the pre-decoding oligonucleotides hybridized to their targets throughout the assay, our method has increased analysis accuracy and decreased assay time and cost. (vi) With each transcript stained in every cycle, this SFO-based approach avoids the false positive and false negative signals generated by the manually selected detection thresholds.

The number of RNA species that can be quantified using this SFO-based approach depends on two factors: the number of hybridization cycles and the number of different fluorophores used in each cycle. As we have demonstrated, at least eight hybridization cycles with high analysis accuracy can be carried out in the same set of cells. And it is well-established that hundreds of thousands of oligonucleotides can be prepared cost-effectively by massively parallel synthesis on a microarray slide (Murgha et al., 2014). Thus, further implementation of the SFO-based approach with four classical fluorophores applied in each cycle will potentially enable the whole transcriptome to be profiled using the 65, 536 (4^8^) distinct fluorophore sequences. Additionally, multispectral fluorophores (Dai et al., 2011; Guo et al., 2011; Wang et al., 2012) coupled with the hyperspectral imaging (Garini et al., 2006) can be applied to allow more fluorophores to be distinguished and applied in each hybridization cycle. In this way, the cycle number together with the assay time can be further reduced. Furthermore, following the RNA profiling by this SFO-based approach, the nuclear and cellular membranes can be counterstained using nuclear staining dyes (such as DAPI) and fluorescent antibodies targeting membrane proteins (such as E cadherin), respectively. With individual cells precisely segmented by this counterstaining approach, the SFO-based approach will allow RNA analysis in single cells of intact tissues. Finally, the combination of this SFO-based approach with multiplexed *in situ* protein analysis technologies (Bodenmiller, 2016; Mondal et al., 2017, in press) will enable the comprehensive and integrated RNA and protein profiling in single cells *in situ*. This molecular imaging platform will bring new insights into systems biology, signaling network regulation, molecular diagnosis and cellular targeted therapy.

## Author contributions

LX and JG designed the experiments. LX performed the experiments. LX and JG analyzed the data and wrote the manuscript.

### Conflict of interest statement

The authors declare that the research was conducted in the absence of any commercial or financial relationships that could be construed as a potential conflict of interest.

## Supplementary material

Table S1 Sequences of the pre-decoding probes, decoding probes, eraser oligonucleotide and SFO-orthogonal oligonucleotide.
